# Functional Redundancy and Ecological Innovation Shape the Circulation of Tick-Transmitted Pathogens

**DOI:** 10.3389/fcimb.2017.00234

**Published:** 2017-05-31

**Authors:** Agustín Estrada-Peña, José de la Fuente, Alejandro Cabezas-Cruz

**Affiliations:** ^1^Faculty of Veterinary Medicine, University of ZaragozaZaragoza, Spain; ^2^SaBio. Instituto de Investigación en Recursos Cinegéticos IREC-CSIC-UCLM-JCCMCiudad Real, Spain; ^3^Department of Veterinary Pathobiology, Center for Veterinary Health Sciences, Oklahoma State UniversityStillwater, OK, United States; ^4^UMR BIPAR, Animal Health Laboratory, Institut National de la Recherche Agronomique, ANSES, ENVAMaisons Alfort, France; ^5^Faculty of Science, University of South BohemiaBudejovice, Czechia; ^6^Biology Centre, Institute of Parasitology, Czech Academy of SciencesCeske Budejovice, Czechia

**Keywords:** networks, ticks, tick-borne pathogens, communities

## Abstract

Ticks are vectors of pathogens affecting human and animal health worldwide. Nevertheless, the ecological and evolutionary interactions between ticks, hosts, and pathogens are largely unknown. Here, we integrated a framework to evaluate the associations of the tick *Ixodes ricinus* with its hosts and environmental niches that impact pathogen circulation. The analysis of tick-hosts association suggested that mammals and lizards were the ancestral hosts of this tick species, and that a leap to Aves occurred around 120 M years ago. The signature of the environmental variables over the host's phylogeny revealed the existence of two clades of vertebrates diverging along a temperature and vegetation split. This is a robust proof that the tick probably experienced a colonization of new niches by adapting to a large set of new hosts, Aves. Interestingly, the colonization of Aves as hosts did not increase significantly the ecological niche of *I. ricinus*, but remarkably Aves are super-spreaders of pathogens. The disparate contribution of Aves to the tick-host-pathogen networks revealed that *I. ricinus* evolved to maximize habitat overlap with some hosts that are super-spreaders of pathogens. These results supported the hypothesis that large host networks are not a requirement of tick survival but pathogen circulation. The biological cost of tick adaptation to non-optimal environmental conditions might be balanced by molecular mechanisms triggered by the pathogens that we have only begun to understand.

## Introduction

Communities are fundamental units of ecological information. They elucidate the interactions among species cohabiting within a defined area and describe changes in species composition and resilience after a disturbance (Christian et al., 2015). Metacommunity theory is a relatively recent development in community ecology (Leibold et al., 2004; Holyoak et al., 2005). The basic postulates of this framework posit local communities are interconnected by the processes of dispersal and extinction (Leibold et al., 2004). The species composition of the local communities is determined by both these regional processes and the local interactions that determine habitat suitability (Holyoak et al., 2005; Leibold and McPeek, 2006; Mihaljevic, 2012). The concepts of the ecological community have been scarcely applied to host-tick systems, in which acquisition of parasites occurs mostly via horizontal transmission (Krasnov et al., 2010). Ecological community approaches could dramatically enhance our understanding of complex pathogen circulation systems that include several vectors and hosts (Estrada-Peña et al., 2015). In addition, since colonization of new habitats is considered to be a frequent event in ectoparasites evolution (Krasnov et al., 2010), we hypothesize that new habitat colonization during tick evolution might have played a especial role in pathogen spread and circulation. The communities of vector-borne pathogens include a panoply of pathogenic microorganisms that circulate through the bite of a competent vector and remain in permanent foci due to the presence of reservoir hosts. The interest in these pathogenic organisms has raised in the last decade due to the increase in the incidence of human diseases related with them (de la Fuente and Estrada-Peña, 2012; Estrada-Peña and de la Fuente, 2014). Ticks are versatile arthropod vectors capable of transmitting the broadest spectrum of pathogens to vertebrates (Jongejan and Uilenberg, 2004). The community approach offers the potential to explore the interactive roles of joint evolutionary history between ticks and their hosts, the impact of abiotic environment, and the evolutionary pressure on associated pathogens. However, this approach is in its infancy, despite some studies focusing on interactions between ectoparasites and their hosts at the local or regional scale (Lindgren et al., 2000; Ostfeld and Keesing, 2000; Keesing et al., 2006, 2010; Jaenson and Lindgren, 2011). Tools such as phylogeography and evolutionary adaptations of the species relationships can be integrated into a framework to quantify environmental and biological factors governing the structure of complex communities of multiple hosts, vectors, and pathogens. These tools have never been jointly applied to the understanding of co-evolution and relationships among the members of a community of tick-transmitted pathogens.

Here, we elaborate on a prominent tick species, *Ixodes ricinus*, that displays complex ecological interactions. This tick species is an interesting model to study the association between tick-host-pathogen communities and the environment because it has a wide distribution in temperate Europe, infest hundreds of vertebrate host species, and support the circulation of several pathogens (Estrada-Peña et al., 2006; Medlock et al., 2013). We explicitly explored how the hosts exploited by this tick result in a functional redundancy that improves the circulation of pathogens. Our results showed that by accessing hosts that are pathogen super-spreaders, ticks occupied non-optimal environments. This is sustainable for the tick only if the cost associated with the colonization of non-optimal environments is balanced by benefits provided by pathogens infection (Cabezas-Cruz et al., 2017).

## Methods

### Background

The methods below refer to the following steps: (i) the collection of association of the metacommunity of ticks, host, and pathogens for the literature, (ii) the construction of the phylogenetic relationships of the hosts of the tick or reservoirs of pathogens, (iii) the construction of the environmental niche of tick and hosts to check for environmental signature in the previous phylogenetic tree, and (iv) the building of the network of ticks and hosts to infer epidemiological relationships. Definitions of the most important terms of this framework are included in Box 1.

Box 1 **Definitions of terms used in this paper.**

**Environmental niche**: The range of abiotic conditions (mainly climate, also known as **environmental variables**) under which an organism can persist. In example, high and low temperature restrict the distribution of living beings. The plot of the recorded distribution of an organism against the main abiotic variables can help to describe its environmental niche. Using a phylogenetic tree of organisms, it is possible to track the gradient of environmental variables to which each organism is associated, known as **environmental signature**.
**Pagel's λ**: It is an index that allows to correlate the environmental variables (or other traits) to a phylogeny. When λ equals 1, the structure of the phylogeny alone can explain changes in environmental niche and therefore environmental variables show a strong phylogenetic signal. On the other hand, when λ equals 0, the phylogeny alone is not able to explain that evolution. The exploration of the phylogenetic signature of environmental variables on a phylogeny tracks if groups of genetically related hosts prefer the same portions of the environmental niche.
**Metacommunity**: An ecological metacommunity is a set of interacting communities of interacting organisms. In this study, we used the term to describe the communities of ticks, vertebrates and tick-transmitted pathogens. Vertebrates interact in the system being hosts of the ticks or reservoirs of the pathogens, ticks are the vectors of the circulating pathogens.
**Network**: An ecological network is a construct that represents biotic interactions between organisms. These organisms are commonly referred to as “nodes,” which interact through “links.” The relationships between interacting organisms provide indexes from which the strength of the associations or the role of organisms in the context of the network can be measured.
**Authority**: The nodes of a network have different indexes that give an idea of its importance in the context of the interacting organisms. The terms “authority” and “hub” are two of these indexes. Nodes with high Authority are nodes which are pointed to by important nodes, in fact, by nodes with high Hub scores. And the latter obtain their high Hub scores by pointing to good Authority nodes. In short: Hubs point, and Authorities are pointed to. The ecological meaning for our application is that i.e., a vertebrate that acts as reservoir of several species of pathogens, or on which the focal tick has been repeatedly recorded, while have a high Authority. It is a measure of the relative importance of the vertebrate (or other organisms) in the network. In the calculation of Authority, the importance of the Hubs is not considered.
**PageRank**: It is another measure of the importance of a single node of the network, that considers the relative importance of the nodes pointing to it. Therefore, a node with a high PageRank is not only linked by many nodes, but with nodes that have also a high relative importance in the network. The ecological meaning is that i.e., a vertebrate will have a high PageRank if nodes of prominent species of pathogens are tightly interacting with it. The difference with Authority is that the importance of the neighbor nodes is considered. Both indexes, Authority and PageRank are complementary.
**Weighted Clustering Coefficient**: Evidence suggests that in most networks, nodes tend to create tight groups characterized by a relatively high density of ties, which is greater than the average probability of a tie randomly established between two nodes. The Weighted Clustering Coefficient measures the degree to which nodes in a graph tend to cluster together. The meaning in our application is that nodes with a high value of centrality will have a tendency to tightly cluster with others, meaning for an ecological interaction that is higher than with other nodes of the network.

### Literature data collection

Data on specific associations of *I. ricinus* and its hosts are the backbone of this study. Data on pairs of associations among ticks, vertebrates, and pathogens were compiled from a literature review focused on the Western Palearctic. A “record” is a pairwise combination of “pathogen and tick,” “tick and vertebrate” or “pathogen and vertebrate” at one site. The pathogen can be associated to either the vertebrate or to the tick, while the tick is only associated with the vertebrate. The literature review was based on journals searchable in Thomson Reuters, Scopus, and PubMed.

We performed a deliberately relaxed query, including only the name “*I. ricinus*,” to manually select the papers reporting ecological information after a critical evaluation of the abstract. The obtained papers were included in the dataset only if adequate information about the host, tick and/or pathogen was available. We purposely removed every report concerning livestock, because they are recognized as accidental hosts, generating spurious information that distorts the natural relationships of the metacommunity of tick-borne pathogens (Estrada-Peña et al., 2015). The literature review included reports published during the period 1970 to December, 2015. As far as possible, the scientific names of pathogens were updated to include the most recent and accepted ones. When not possible (i.e., the complex of species of *Borrelia burgdorferi*, whose specific names changed radically in the last years) it has been included as a generic name (in the example, *Borrelia burgdorferi* sensu lato) to reflect the relationships but without reliability of the name of the pathogen.

### Phylogenetic relationships of hosts

**Figure 1:**
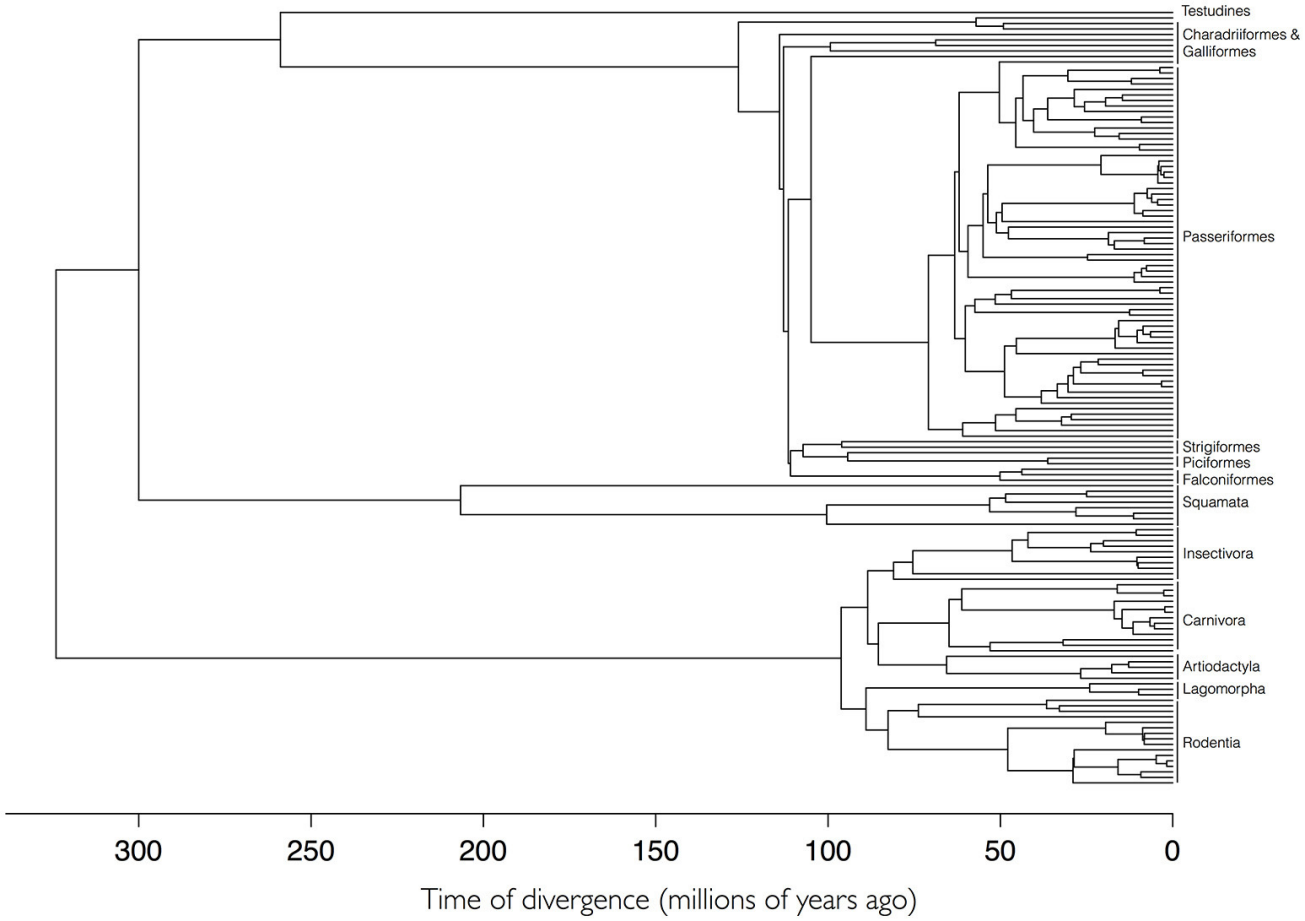
The phylogenetic tree of 168 species of hosts of *Ixodes ricinus* based on a supertree of Tetrapoda of Western Palearctic. Higher taxonomical categories of hosts are included at the tips of the tree. Supplementary Figure S1 provides a high-resolution version of this figure with the names of species at the tips of the tree.

A total of 168 species of vertebrates were recorded as hosts for *I. ricinus*. We used a previously published, dated supertree of Tetrapoda of the Western Palearctic (Roquet et al., 2014). The complete details for the construction of the supertree have been published and extensively contrasted (Roquet et al., 2014). The tree of Tetrapoda was pruned to accommodate only the hosts of *I. ricinus* using the package ape (Paradis et al., 2004) for the R programming environment (R Core Team, 2014). The complete phylogenetic tree of the hosts of *I. ricinus* is available in newick format as Figure 1, Supplementary Table S1, and Supplementary Figure S1.

### Data on environmental variables and calculation of niche dimensions of ticks and hosts

We wanted to track the environmental niche in which each species of hosts prevails and how the environmental niche of the tick and its hosts overlap. This is commonly captured by running models that measure the distribution of the tick and each host species in the environmental niche. We used a previously developed global dataset of environmental variables (Estrada-Peña et al., 2014) based on the transformation by harmonic regression of monthly data derived from the MODIS series of satellites at a nominal resolution of 0.05°. The dataset includes day temperature (LSTD) and the Normalized Difference Vegetation Index (NDVI), an index of vegetation vigor, which were obtained for the period 2001–2015. We retained three variables for LSTD (LSTD1–LSTD3) and three for NDVI (NDVI1–NDVI3) that explain the annual average and the slope (seasonality) in spring and autumn, respectively. The ability of the dataset to capture the environmental niche of organisms has been already demonstrated (Estrada-Peña et al., 2014).

We obtained pairs of coordinates of every species of host for the tick querying the Global Biological Information Facility (GBIF) (Garcia-Rosello et al., 2014). Approximately 11 million records were obtained for 203 unique species. We retained a set of 142 hosts for which phylogenetic information and reliable distribution data (at least 100 geo-referenced records) were simultaneously available. We independently trained environmental suitability models of each species of host using the niche modeling program MaxEnt (Phillips et al., 2006). MaxEnt was chosen because it demonstrated robust model performance compared to other modeling algorithms when presence-only data is available. We used the lineal and quadratic features with a maximum number of 10,000 background points and 70% of points for training purposes, using cross-validation to compare the resulting models. The regularization multiplier was set to 1. Each model was replicated 100 times using the cross-validation function in MaxEnt to partition the data into replicate folds, with each fold being used in turn to test the model. The aims of model building were to: (i) identify the host's environmental niche (Elith et al., 2011), (ii) reconstruct the phylogenetic signature of the environmental variables in the phylogenetic tree of hosts, and (iii) calculate the niche overlap between the tick and each host, following published approaches (Warren et al., 2010). The niche of *I. ricinus* was calculated using the same methods and a set of more than 8,000 occurrence records of the species with reliable coordinates (Estrada-Peña and de La Fuente, 2016).

### Inferring the signature of environmental traits in the host phylogeny

We wanted to track the signature of the environmental variables on the phylogenetic tree of hosts for *I. ricinus* to check if the environmental niche is conserved along the phylogeny of hosts. We could therefore conclude that the tick exploits hosts that share environmental conditions because they are genetically related. We treated environmental niche of species as evolving traits whose evolutionary history can be reconstructed through phylogenetic analysis, calculating a potential niche occupancy for ranges of the environmental covariates. The original supertree of Tetrapoda (Roquet et al., 2014) was pruned to retain the species of vertebrates exploited as hosts by *I. ricinus* as obtained from the literature search mentioned above, resulting in a sub-phylogeny of 142 vertebrate species. We used Pagel's λ (see Box 1) to measure the phylogenetic signal of the environmental variables along the phylogenetic tree (Munkemuller et al., 2012) as implemented in *phytools* (Revell, 2012) for R Core Team (2014). We calculated Pagel's λ separately for each of the six environmental variables (LSTD1-LSTD3 and NDVI1-NDVI3) on the phylogenetic tree of hosts for *I. ricinus*.

### Building the network of hosts of *I. ricinus* and transmitted pathogens

The analyses before produced an estimation of the niche overlap between *I. ricinus* and its hosts as well as the correlation between the phylogenetic tree and the environmental variables. The last step of our framework aims to evaluate the impact of each host on the circulation of the pathogens.

We used a dataset on reported relationships between ticks, hosts, and pathogens to develop a network of biotic connections (Estrada-Peña and de La Fuente, 2016) in terms of “who is a parasite of whom” and then obtain conclusions on the effects of that structure on the circulation of pathogens. In host-parasite networks (see Box 1), nodes represent “cargos” that are linked to “carriers.” Thus, the network is directed: each edge links a pathogen “to” a vertebrate or a tick, or a tick to a vertebrate. “Cargos” are thus the pathogens or the ticks, “carriers” are the ticks (for pathogens) or the vertebrates (as hosts for ticks or reservoirs for pathogens).

Centrality measures in ecological networks detect high-ranking nodes in the network (Blondel et al., 2008; Jacomy et al., 2014). To be central, a carrier is infected by many species of cargos that infect many other carriers in the network. The hosts with the greatest centrality are super-spreaders. Authority (Box 1) is an index that provides the importance of a node to which other nodes are connected (Holland and Leinhardt, 1971; Kourtellis et al., 2013). This is complimentary to PageRank (Box 1), an index that assigns a rank to nodes based on the importance of the other nodes to which it is linked (Holland and Leinhardt, 1971). The Weighted Clustering Coefficient is a measure of the degree to which nodes in a graph tend to cluster together (Holland and Leinhardt, 1971; Watts and Strogatz, 1998; Kourtellis et al., 2013). The Weighted Clustering Coefficient (Box 1) expresses the statistical level of cohesiveness measuring the global density of interconnected nodes in the network. Network computations were carried out using *igraph* (Csardi and Nepusz, 2006) for R Core Team (2014), the Louvaine clustering algorithm (Blondel et al., 2008) and the ForceAtlas2 algorithm for displaying the network (Jacomy et al., 2014).

## Results

### *Ixodes ricinus* uses hosts with a phylogenetic signature of environmental niche

**Figure 2:**
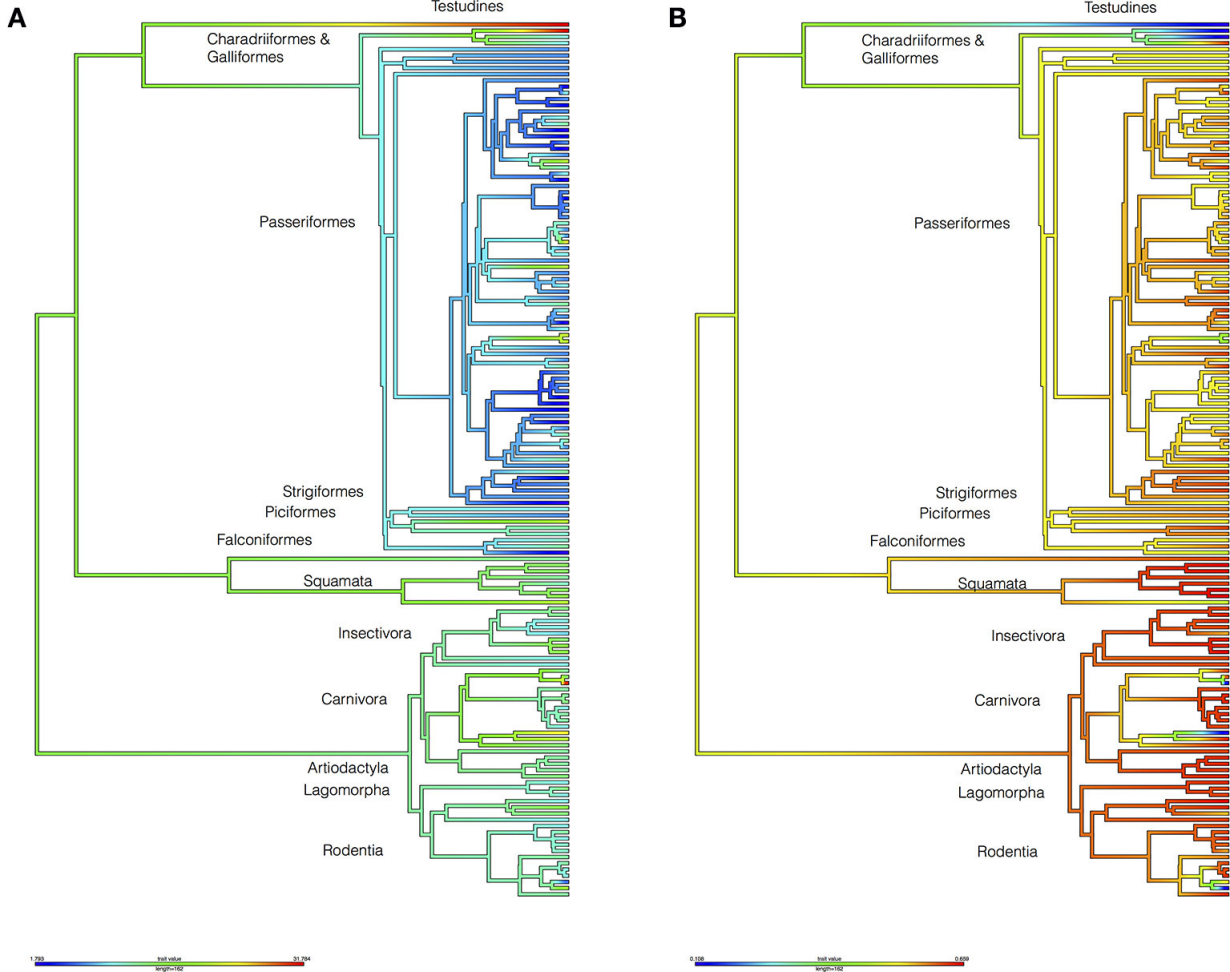
Reconstructions of the environmental niche of the hosts of *I. ricinus*. The figure includes data for Land Surface Temperature (LSTD: **A**) and the Normalized Difference Vegetation Index (NDVI: **B**). The trees were drawn according to the phylogenetic tree in the Figure 1. Values in the legend are degree Celsius **(A)** and NDVI units multiplied by 100 **(B)**. Higher taxonomical categories of hosts are included at the tips of the tree. Supplementary Figure 2 provides a high-resolution version of this figure with the names of species at the tips of the tree.

To test whether the hosts of *I. ricinus* Were significantly associated to environmental variables (i.e., LSTD and NDVI, see Methods), we built a host phylogenetic tree and measured the phylogenetic signal of the environmental signature (see Box 1 for definitions). A strong signature was found along the phylogeny of *I. ricinus* hosts (Figure 2, Supplementary Figure S2). Pagel's λ (see Box 1) was 0.69 (*p* = 3.86E-8) for the mean of LSTD and 0.66 (*p* = 0.0002) for the mean of NDVI. Other variables tested, like seasonality of LSTD and NDVI, lack phylogenetic signature (λ < 0.3). Most non-Aves hosts of *I. ricinus* were linked to warmer portions of the niche, whereas almost all species in Aves colonized a colder portion of the niche. All of the non-Aves vertebrates colonized the greenest portion of the environmental niche, whereas most Aves species were restricted to intermediate values of NDVI. Notably, non-Aves hosts evolved earlier than Aves. The fact that the older hosts occupied the warmer portions of the niche suggests that the ancestral state of the phylogenetic signature of the temperature for the hosts of *I. ricinus* was the warmer portions of the niche. The colonization of a colder niche was inferred to be a derived state according to the dating of the Tetrapoda genetic tree. The ecological explanation of these results is that *I. ricinus* is ecologically tied to two large clusters of hosts. The clear dichotomy in the temperature range (as opposed to a gradient) colonized by both clusters of vertebrates is highly suggestive of a host split in which *I. ricinus* accessed a new environmental niche while simultaneously adapting to a new set of hosts. Interestingly, vegetation (and therefore humidity) seems to play a secondary but still significant role in that split of hosts. The niche overlap between *I. ricinus* and its hosts produced a low phylogenetic signal (λ = 0.188, *p* = 0.337). The ecological interpretation is that the amount of niche shared between *I. ricinus* and its hosts is unrelated to the genetic relationships of the vertebrates: the tick does not exploit species of vertebrates that are closely related to gain a wider environmental niche.

**Figure 3:**
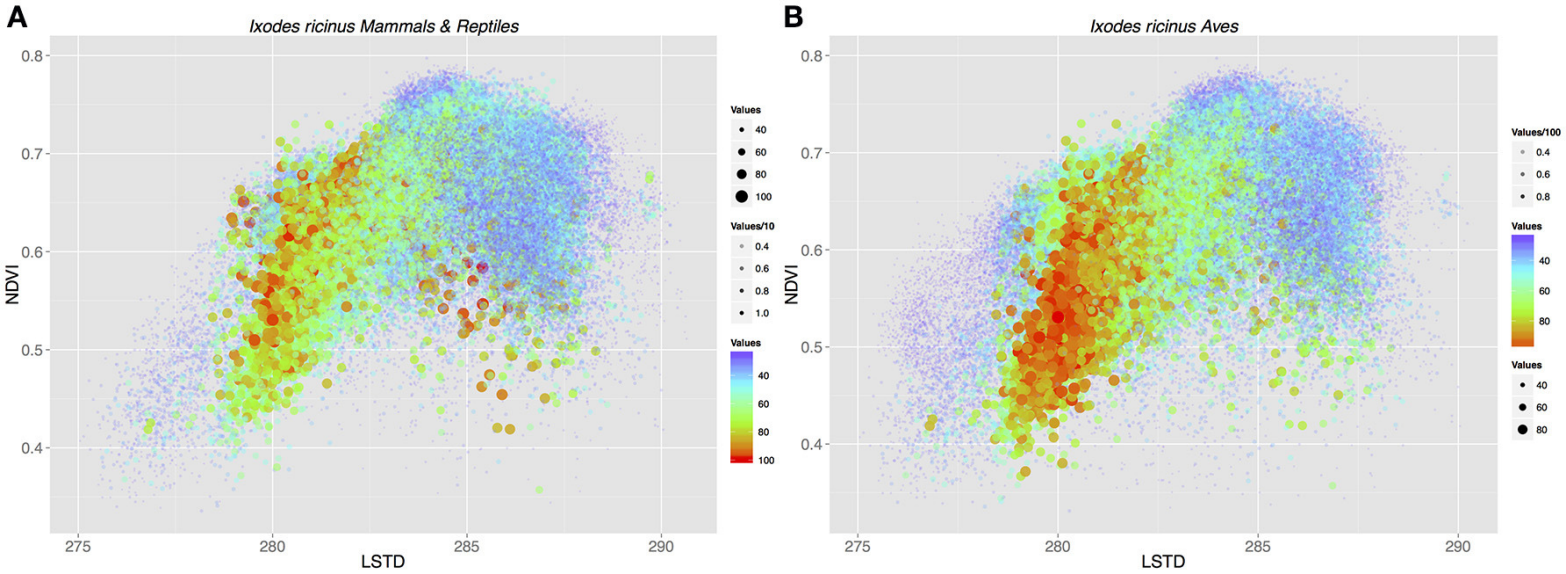
The suitability of the environmental space formed by the intersection of average annual land surface temperature (LSTD) and average annual Normalized Difference Vegetation Index (NDVI) for *I. ricinus*. Dots are the product of the suitability of a specific portion of the abiotic niche for the tick according to environmental traits, the habitat overlap with hosts at that specific point, and the environmental suitability for the host(s) available at that specific point. Because of the large number of points displayed (>6 × 10^6^), color, size, and transparency are used together to improve the readability of the charts. **(A)** The niche suitability was calculated with mammals and lizards as the only available hosts. **(B)** Birds were added to calculations of niche suitability for *I. ricinus*.

To test the contribution of different host groups to the total environmental niche available to *I. ricinus*, we modeled the environmental suitability of this tick across different environmental conditions considering that only a specific set of vertebrates was exploited. Calculations were done considering different environmental parameters of temperature (LSTD) and humidity (NDVI) and including only “mammals+reptiles” or “mammals+reptiles+birds.” Figure 3 shows the environmental suitability for *I. ricinus* in the niche described by the mean LSTD and NDVI. The inclusion of Aves in calculations resulted in higher suitability at the range of low temperature. However, the net gain in habitat including birds as hosts was only of 8.2%. These results showed that the adaptation of *I. ricinus* to 85 species of Aves, hypothesized to be a derived state, is not sufficiently explained by environmental factors (i.e., temperature and humidity) crucial for tick physiology. In other words, even if the tick could exploit more hosts (Aves) across a wider environmental gradient (colder temperatures), the net gain in niche is too small to confer an adaptive advantage to the tick. This lead us to the hypothesis that the colonization of new habitats by *I. ricinus* might have played a especial role in pathogen spread and circulation.

### A network of connections between ticks, hosts, and pathogens reveals the structure of the biotic relationships of *I. ricinus*

To evaluate the effect of the colonization of Aves and colder regions by *I. ricinus* in pathogen spread and circulation, we developed a network of connections between ticks, hosts, and pathogens. This is a directed network where ticks were recorded on hosts or pathogens detected on vertebrates and/or ticks. The network contains records of vertebrate hosts and transmitted pathogens associated to *I. ricinus*. To gain a complete view of the network, we also included other tick species recorded on the same species of vertebrates. The complete network has 379 nodes (organisms) and 1020 links (connections) among them (Supplementary Figure S3). We identified seven communities of organisms that are more associated with each other than with other members of the network. *I. ricinus* is the pivot of a large cluster in which other tick species (*Ixodes redikorzevi, Ixodes acuminatus, Ixodes persulcatus*, and *Haemaphysalis concinna*) are included. In the network, *I. ricinus* links 160 host species and 19 pathogens. *I. ricinus* is linked to 48 families and 86 genera of vertebrates. Table 1 summarizes the features of centrality regarding the cluster of *I. ricinus*, higher centrality values meaning for a more prominent role of these nodes in supporting the network. Passeriformes have twice the Authority of Rodentia, three times higher PageRank, and twice the Weighted Clustering Coefficient. The ecological translation is that the Passeriformes contribute more to the resilience of the tick and allow better circulation of the transmitted pathogens.

**Table 1:** Centrality indexes of the hosts forming the network of vertebrates and transmitted pathogens in which *Ixodes ricinus* acts as a carrier.

	**Order of hosts**	**#Species of hosts**	**Authority**	**PageRank**	**Weighted clustering coefficient**
Aves	Accipitriformes	3	0.00739	0.00954	0.0000
Aves	Charadriiformes	2	0.00422	0.00632	0.0000
Aves	Coraciiformes	1	0.00211	0.00316	0.0000
Aves	Falconiformes	1	0.00528	0.00451	0.0000
Aves	Galliformes	3	0.01056	0.01228	0.0000
Aves	Gruiformes	1	0.00211	0.00316	0.0000
Aves	Passeriformes	75	**0.32154**	**0.26377**	**5.6237**
Aves	Piciformes	2	0.00422	0.00632	0.0000
Aves	Strigiformes	2	0.00528	0.00680	0.0000
Mammalia	Rodentia	18	**0.15470**	**0.08599**	**2.1718**
Mammalia	Artiodactyla	9	0.09715	0.05254	1.1443
Mammalia	Carnivora	14	0.07497	0.06055	0.5935
Mammalia	Erinaceomorpha	3	0.03273	0.01771	0.1536
Mammalia	Lagomoprha	4	0.02746	0.01931	0.0463
Mammalia	Soricomorpha	9	0.03485	0.03064	0.6470
Reptilia	Testudines	1	0.00528	0.00619	0.0000
Reptilia	Squamata	12	0.05491	0.04545	0.5012
	Total Aves	90	0.36272	0.31584	5.6237
	Total Mammalia	57	0.42186	0.26674	4.7565
	Total Reptilia	13	0.06019	0.05164	0.5012

*Numbers in bold refer to the highly prominent hosts in the network of transmitted pathogens*.

**Figure 4:**
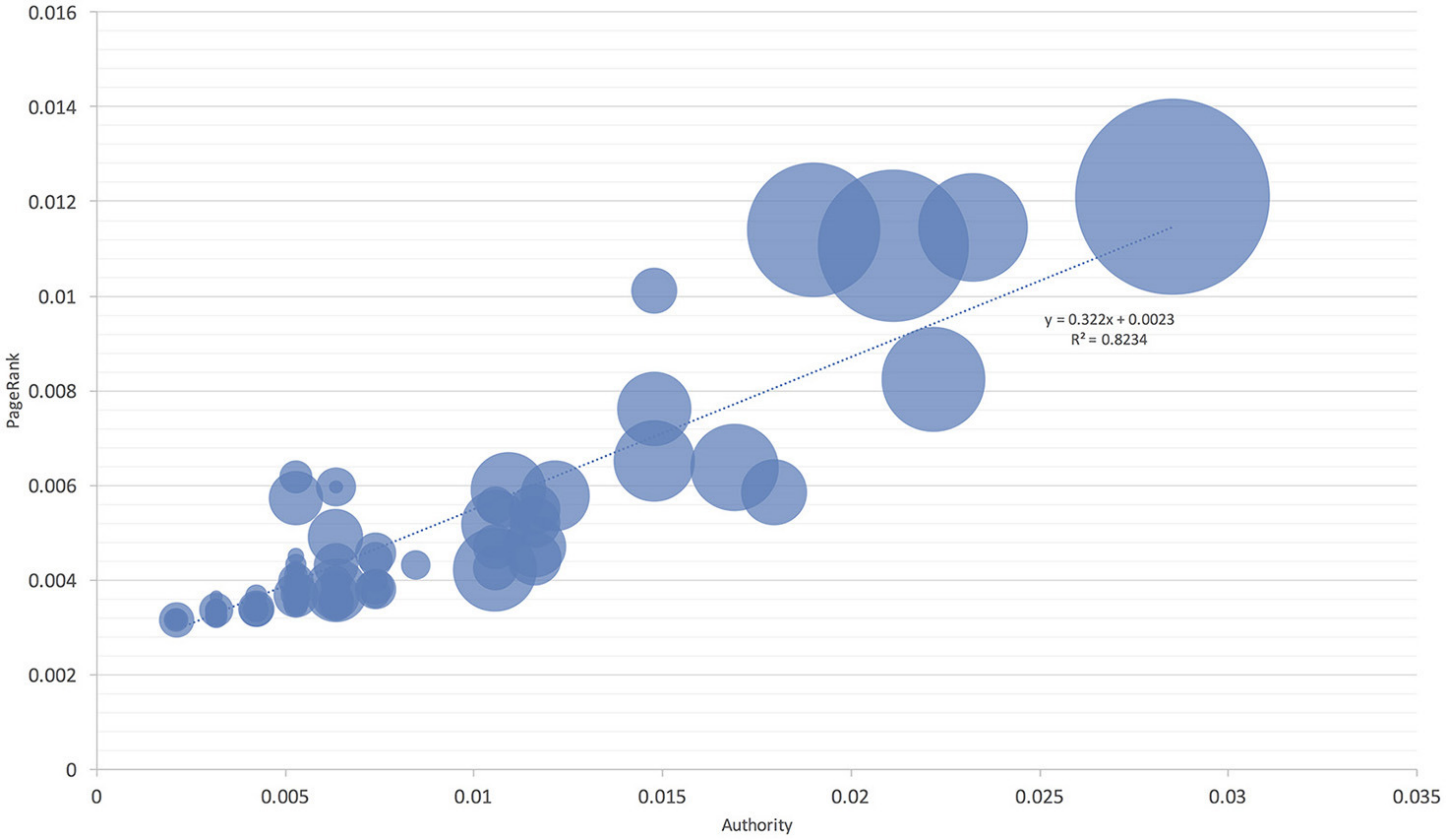
The proportion of niche overlapping between *I. ricinus* and its hosts. The area of each circle is proportional to niche overlap and related to two measures of host centrality, namely the Authority and PageRank. The chart includes all reported hosts for the tick. The line shows the regression between the niche overlap, Authority, and PageRank.

**Figure 5:**
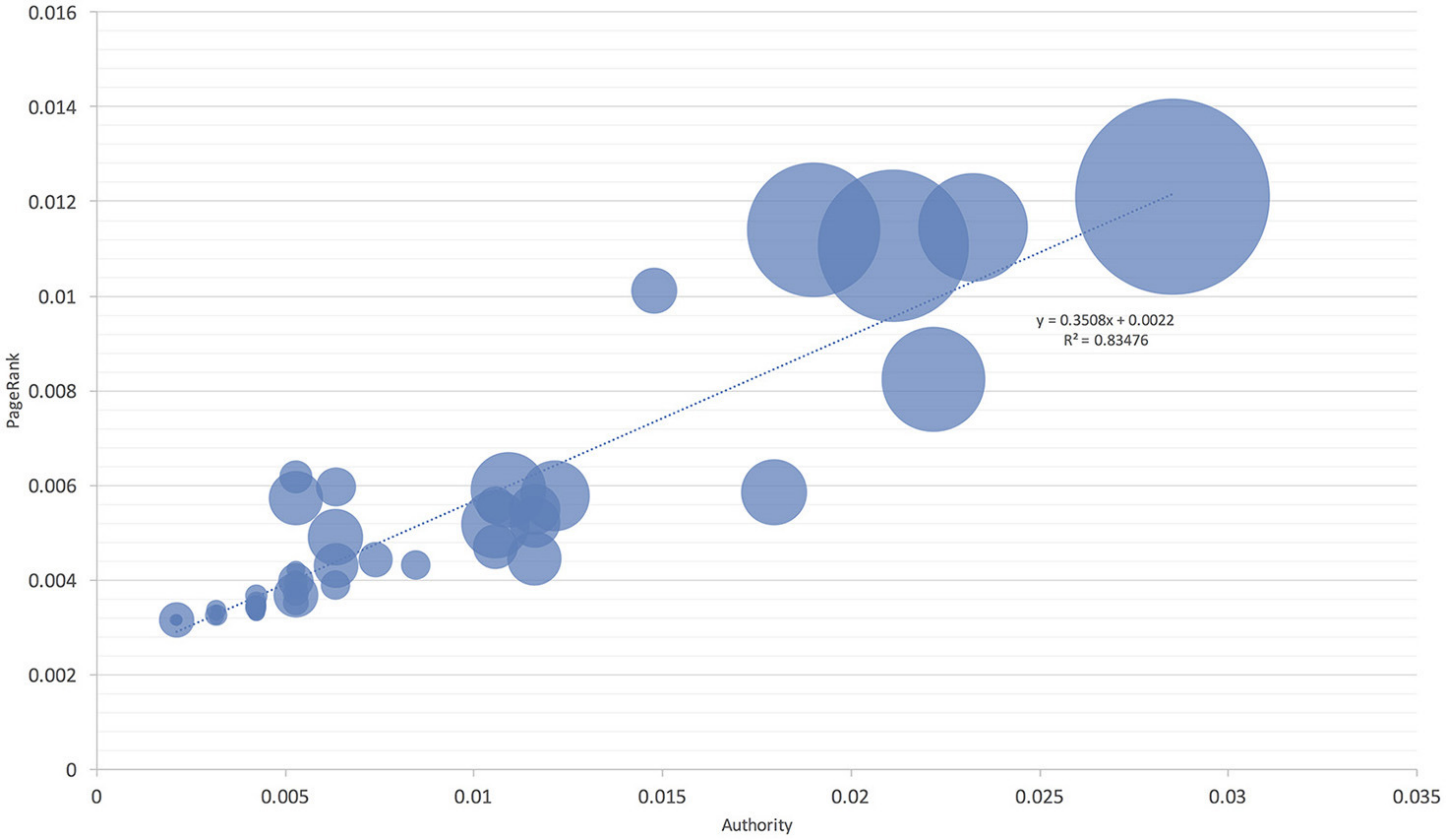
The proportion of niche overlapping between *I. ricinus* and its hosts without Aves. The area of each circle is proportional to niche overlap and related to two measures of host centrality, namely the Authority and PageRank. The chart excludes Aves as hosts for the tick in the calculations and is intended to show the lack of habitat overlap at a large range of the Authority-PageRank gradient. The line shows the regression between the habitat overlap, Authority, and PageRank.

These observations were further confirmed by comparing the niche overlap of ticks and hosts according to the network-derived properties (Figures 4, 5). *I. ricinus* shares higher fractions of the environmental niche with the hosts that have greater measures of centrality in the network. Therefore, *I. ricinus* maximizes the niche overlap with the hosts that have a more prominent role in the circulation of the pathogens, independently of their phylogenetic relationships. Since the maximization of the niche sharing improves the circulation of the tick and transmitted pathogens, removing birds from these calculations results in severe reduction of large areas of suitability of the tick and pathogens at intermediate positions of the gradient of centrality measures. The results demonstrated that the use of Aves as hosts by *I. ricinus* (and the gain of a new niche for the tick) shapes the persistence and resilience of the transmitted pathogens.

## Discussion

This study captured the structural details of a community of pathogens transmitted by a tick and circulating through different clades of vertebrates. It is the development of a proof-of-concept (Estrada-Peña et al., 2015) intended to accommodate the structure of a network to the biotic relationships between parasites and hosts, and combines techniques from phylogenetics and niche modeling. The different parts of this study were derived from distinct disciplines with their own intrinsic assumptions, limitations, and requirements. Therefore, integration is critical for the success of this methodological approach. Significant challenges are apparent because these datasets are commonly affected by bias due to the abundance of common hosts or interest in a given pathogen. Therefore, the dataset must be weighted adequately to not reflect bias affecting the structure of relationships. We adhered to reliable protocols to reconstruct the climate niches (Warren et al., 2010), extract the basic environmental envelope of both the tick and its hosts (Phillips et al., 2006; Elith et al., 2011), or weight the records of interactions for the network (Gómez et al., 2013; Estrada-Peña et al., 2015). To examine how niches of hosts evolved, ancestral state reconstruction methods were used on a phylogenetic tree of Tetrapoda (Munkemuller et al., 2012; Roquet et al., 2014).

Results revealed that the tick does not exploit vertebrates because they are phylogenetically related. The reconstruction of the ancestral state of the environmental niche of hosts is strongly suggestive of a split in the abiotic niche of the tick, when it adopted several species of Aves as hosts. The event probably occurred around 120 M years ago and seems to not be related to recent glaciations, which are known to shape the refugia for vertebrates (Jaarola et al., 1999; Deffontaine et al., 2005; Kasapidis et al., 2005; Sommer and Nadachowski, 2006; Venditti et al., 2011). From our results, the ancestral hosts for *I. ricinus* are interpreted to be mammals and reptiles. The colder portions of the niche are assumed to be a derived state because Aves evolved later in geological times. However, it is unclear whether the tick or an ancestor was a parasite of an extinct relative of the current hosts, which further speciated, spreading the tick into new ecological niches to which it adapted. In this hypothesis, other tick lineages would have become extinct because of a lack of adaptation to the new set of environmental conditions. The opposite hypothesis is that the tick was a parasite of mammals and reptiles, and changes in climate pushed the tick to contact new niches finding a new set of hosts to exploit. The former hypothesis is host-driven, allowing the tick to adapt to new environmental traits; the latter is driven by environmental traits, with the tick exploiting a new set of hosts that were available under these new conditions. Environmental filtering has been predicted to generate phylogenetic clustering (Jaarola et al., 1999; Deffontaine et al., 2005; Kasapidis et al., 2005; Brooks et al., 2006; Sommer and Nadachowski, 2006; Venditti et al., 2011; Suzán et al., 2015) because closely related host species share similar niches.

Speciation of the *I. ricinus* group could have occurred after the separation of Laurasia. The time during which Passeriformes evolved was an unusually warm geological interval, followed by a long period of colder temperatures lasting until the recent glaciations. These dates overlap well with the estimated split in the temperature traits based on the phylogenetic signature in the dated host tree. These are key events for the tick's ecological innovation that allows the exploitation of new resources or habitats (Heard and Hauser, 1995). Ecological innovation enhances competitive ability, or permits exploitation of new resources, and has commonly been reported to increase the divergence rates of clades of species (Peterson and Holt, 2003). The adoption of a colder niche implies an ecological cost for the tick, because lower temperatures imply adaptations for a longer life cycle with concurrent higher mortality (Estrada-Peña and de la Fuente, 2014). The split to new hosts could also involve a cost in terms of adaptation to the new “molecular environment” of the hosts, such as the evasion of immune responses (de la Fuente et al., 2015). Thus, it is central to this study to address why the tick persisted to circulate in such a diverse community of new hosts and wide range of thermal conditions.

Networks are pervasive across all levels of biological organization (Watts and Strogatz, 1998). These structures have been commonly used to represent food webs (Cattin et al., 2004) or plant-pollinator relationships (Dormann, 2011), and only recently were applied for describing the epidemiological context of ticks, vertebrates, and pathogens (Estrada-Peña et al., 2015). Our results revealed conceptual aspects of the ecological interactions and showed that the host split by the tick has a considerable impact on the transmitted pathogens. *I. ricinus* pivots around a rich array of relationships in nested sub-networks: phylogenetically distant vertebrates enhance the circulation of pathogens by providing a functional redundancy along the environmental gradient. Pathogens, even if restricted to a set of reservoirs (Kurtenbach et al., 1994) would have greater resilience if the vector (i) maximizes the number of interactions with the most central hosts of the network, boosting the circulation of pathogens across the sub-networks, and (ii) exploits many species of hosts established along the gradient of the niche variables.

We hypothesize that the biotic interactions of the tick with the community of vertebrates improve the circulation of the pathogens which are in turn involved in the adaptation of the tick to a wide environmental niche occupied by genetically unrelated hosts. The mechanisms that support the resilience of the tick vector in such diverse environment are far from being well-understood. Work in progress suggests that the co-evolutionary mechanisms of tick-transmitted micro-organisms could benefit the persistence of the tick. In particular, we recently proposed that tick-pathogen associations evolved to form “*intimate epigenetic relationships*” in which the pathogen induces transcriptional reprogramming in infected ticks (Cabezas-Cruz et al., 2017). This will ultimately favor pathogen propagation, but will also select for the most suitable ecological adaptations in the tick vector. These phenotypic and genetic changes may have the potential to be transmitted to the next generation of ticks. As a result, the ecological associations between tick, vertebrates and pathogens would evolve to maximize pathogen circulation in these communities (Estrada-Peña et al., 2015; Cabezas-Cruz et al., 2017).

An interesting example is the intracellular pathogen *Anaplasma phagocytophilum* that manipulates tick protective responses to facilitate infection and preserve tick feeding and vectorial capacity to guarantee the survival of both pathogens and ticks (Neelakanta et al., 2010; Merino et al., 2011; Busby et al., 2012; Ayllón et al., 2015a,b; de la Fuente et al., 2017). These mechanisms include the expression of an antifreeze glycoprotein to increase tick survival at cold temperatures (Neelakanta et al., 2010). It was further showed that improved survival of infected ticks correlated with higher *A. phagocytophilum* infection, therefore providing a direct link between pathogen infection and tick fitness in cold environments (Neelakanta et al., 2010). Heat shock proteins (HSP) are also induced by *A. phagocytophilum* infection and protect ticks from stress and pathogen infection (Busby et al., 2012). The HSP responses help increase tick survival by protecting them from stress and preventing desiccation at high temperatures after enhancing questing speed in order to increase the chances of a tick attaching to a host. Similarly, *A. phagocytophilum* subverts the tick RNA interference response to preserve tick feeding (Ayllón et al., 2015b).

Another example of tick-pathogen association is *Borrelia* spp. In the field, *Borrelia*-infected rodents have higher tick burdens than uninfected rodents (Hanincova et al., 2003; Gassner et al., 2013). Studies have also shown that *Borrelia*-infected ticks are more tolerant to desiccating conditions, which commonly stop questing activity and increase tick mortality (Herrmann et al., 2013). Hosts with high nymphal tick burden have a greater chance of becoming infected with *Borrelia*, and rodents infested with nymphs have higher larval tick burdens than rodents without nymphs (Craine et al., 1995; Bown et al., 2008). The molecular mechanisms involved in this regulation are still unknown, but together they contribute to demonstrate how the cargo rewards the circulation of the infected carriers, which are promoted to find a host earlier than non-infected ticks and infect more vertebrate reservoirs. Additional studies with other tick-borne pathogens and deeper analysis of these co-evolutionary adaptations are necessary before we can generalize these findings (de la Fuente et al., 2017).

In this study, we integrated phylogenetics and network analyses to demonstrate that the diversity of hosts increases the niche available for a tick and promotes the circulation of transmitted-pathogens. *I. ricinus* uses a large assemblage of hosts that are phylogenetically unrelated and that split into different values of environmental conditions with an obvious phylogenetic signature. The tick maximizes the niche overlap with the most important hosts in the network, but is further supported by a profusion of secondary hosts that provide the functional redundancy to the network. We interpreted this finding not only as a way for the tick to persist in a variety of conditions, but also as a strategy to enhance the circulation of pathogens. The data are highly suggestive of a dramatic decrease in the circulation of *I. ricinus*-transmitted pathogens if the dozens of species of birds used as hosts by the tick (interpreted as an acquired event in the geological times) are removed. This is a change of paradigm providing evidence that the functional redundancy of the vertebrates enhances the circulation of transmitted pathogens, even if the net gain of environmental niche is low. Though tick larvae would feed mostly on rodents on the local scale, birds are the stepping stones for disseminating the nymphs and enhance the circulation of the community. These results strongly suggest that pathogens may manipulate ticks to occupy sub-optimal environmental niches. Transmission rates between ticks and vertebrates should be incorporated into the network structure to evaluate the contribution of each host to the system and the regulatory mechanisms operating at each level of complexity. These results have important implications for a deeper understanding of the idiosyncratic factors regulating the prevalence of tick-borne diseases.

## Author contributions

AE designed the work and prepared the figures. AE and AC obtained the results. All authors wrote the manuscript and approved the final version.

### Conflict of interest statement

The authors declare that the research was conducted in the absence of any commercial or financial relationships that could be construed as a potential conflict of interest.

## Supplementary material

Supplementary Figure S1 The phylogenetic tree of 168 species of hosts of *Ixodes ricinus* based on a supertree of Tetrapoda of Western Palearctic. This is a high-resolution version of Figure 1 with the names of the species of hosts included at the tips of the tree.

Supplementary Figure S2 Reconstructions of the environmental niche of the hosts of *I. ricinus*. The figure includes data for Lands Surface Temperature (LSTD: **A**) and the Normalized Difference Vegetation Index (NDVI: **B**). The trees were drawn according to the phylogenetic tree in the Figure 1. Values in the legend are degree Celsius **(A)** and NDVI units multiplied by 100 **(B)**. Higher taxonomical categories of hosts are included at the tips of the tree. This is a high resolution version of Figure 2 with the names of the species of hosts included at the tips of the tree.

Supplementary Figure S3 The network of tick *I. ricinus*, the satellite species of ticks, vertebrate hosts, and transmitted pathogens. Circles are organisms (ticks, hosts, pathogens) and lines are links (interactions) among them. Colors in the figure indicate clusters retrieved by an algorithm that groups together the organisms that are closer than others. Clusters include other species of ticks that are satellite to the focal species because they share hosts. The size of each circle indicates the centrality of the organism. The width of each link is proportional to the strength of the interaction between two given organisms.

Supplementary Table S1 The tree in newick format used to draw the phylogenetic relationships between the vertebrates recorded as hosts of *Ixodes ricinus*.
