# Supplementary figures and images for: Functional Redundancy and Ecological Innovation Shape the Circulation of Tick-Transmitted Pathogens
Source: Front Cell Infect Microbiol. 2017 May 31;7:234. doi: 10.3389/fcimb.2017.00234 (PMC5450623; doi:10.3389/fcimb.2017.00234)

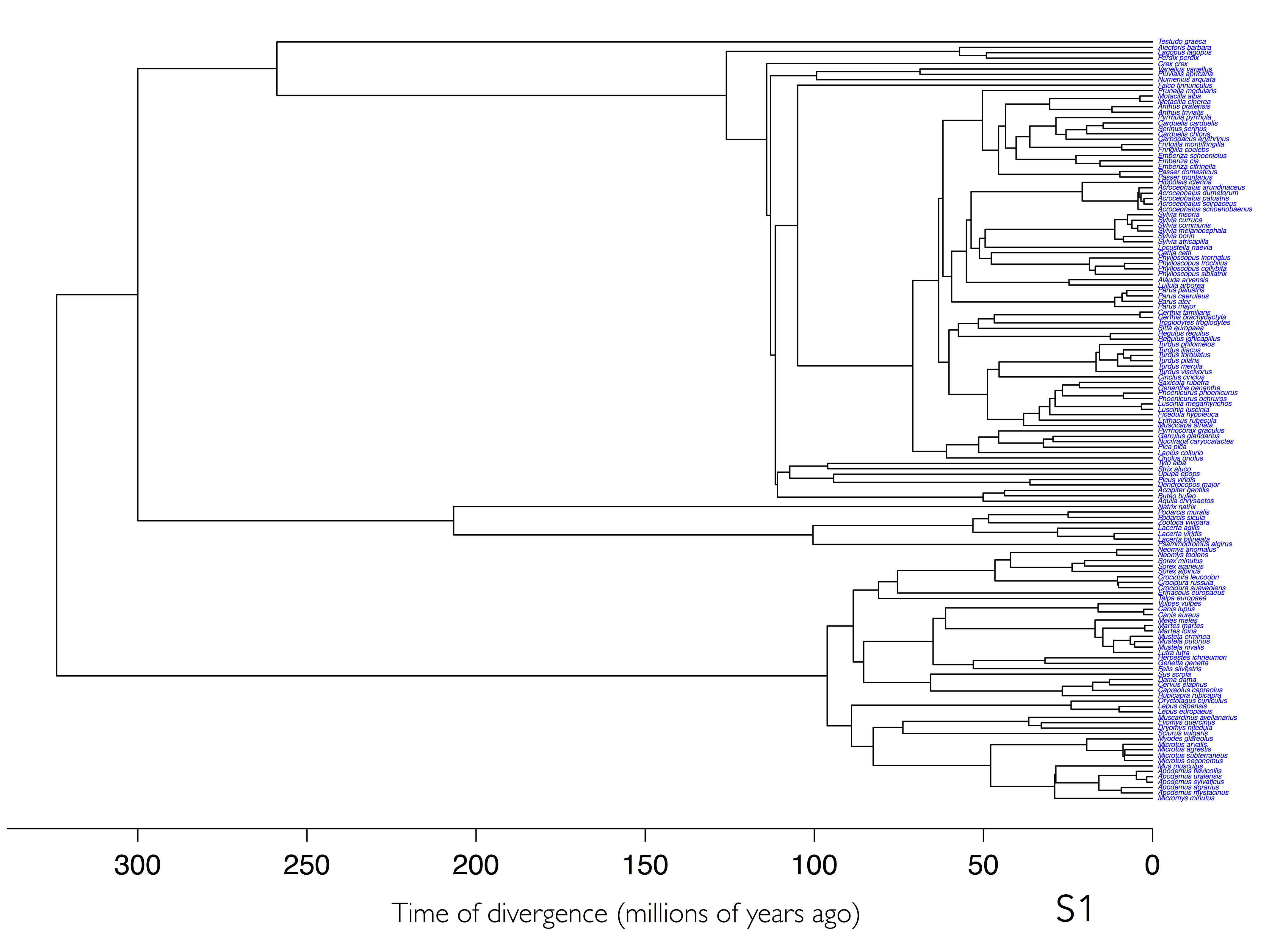

Supplement: Supplementary Figure S1 — The phylogenetic tree of 168 species of hosts of Ixodes ricinus based on a supertree of Tetrapoda of Western Palearctic. This is a high-resolution version of Figure 1 with the names of the species of hosts included at the tips of the tree. [file Image1.TIFF]

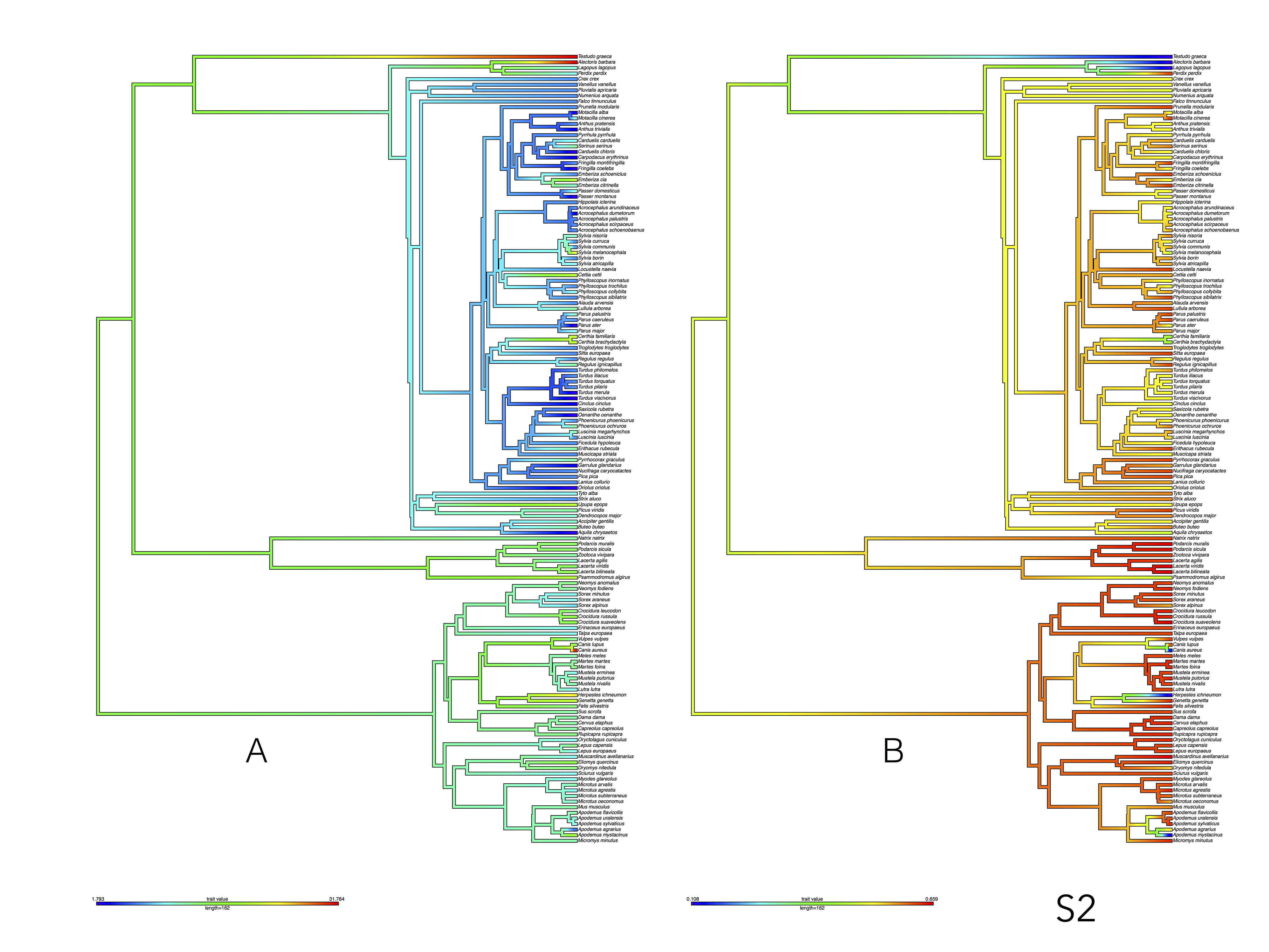

Supplement: Supplementary Figure S2 — Reconstructions of the environmental niche of the hosts of I. ricinus. The figure includes data for Lands Surface Temperature (LSTD: A) and the Normalized Difference Vegetation Index (NDVI: B). The trees were drawn according to the phylogenetic tree in the Figure 1. Values in the legend are degree Celsius (A) and NDVI units multiplied by 100 (B). Higher taxonomical categories of hosts are included at the tips of the tree. This is a high resolution version of Figure 2 with the names of the species of hosts included at the tips of the tree. [file Image2.TIFF]

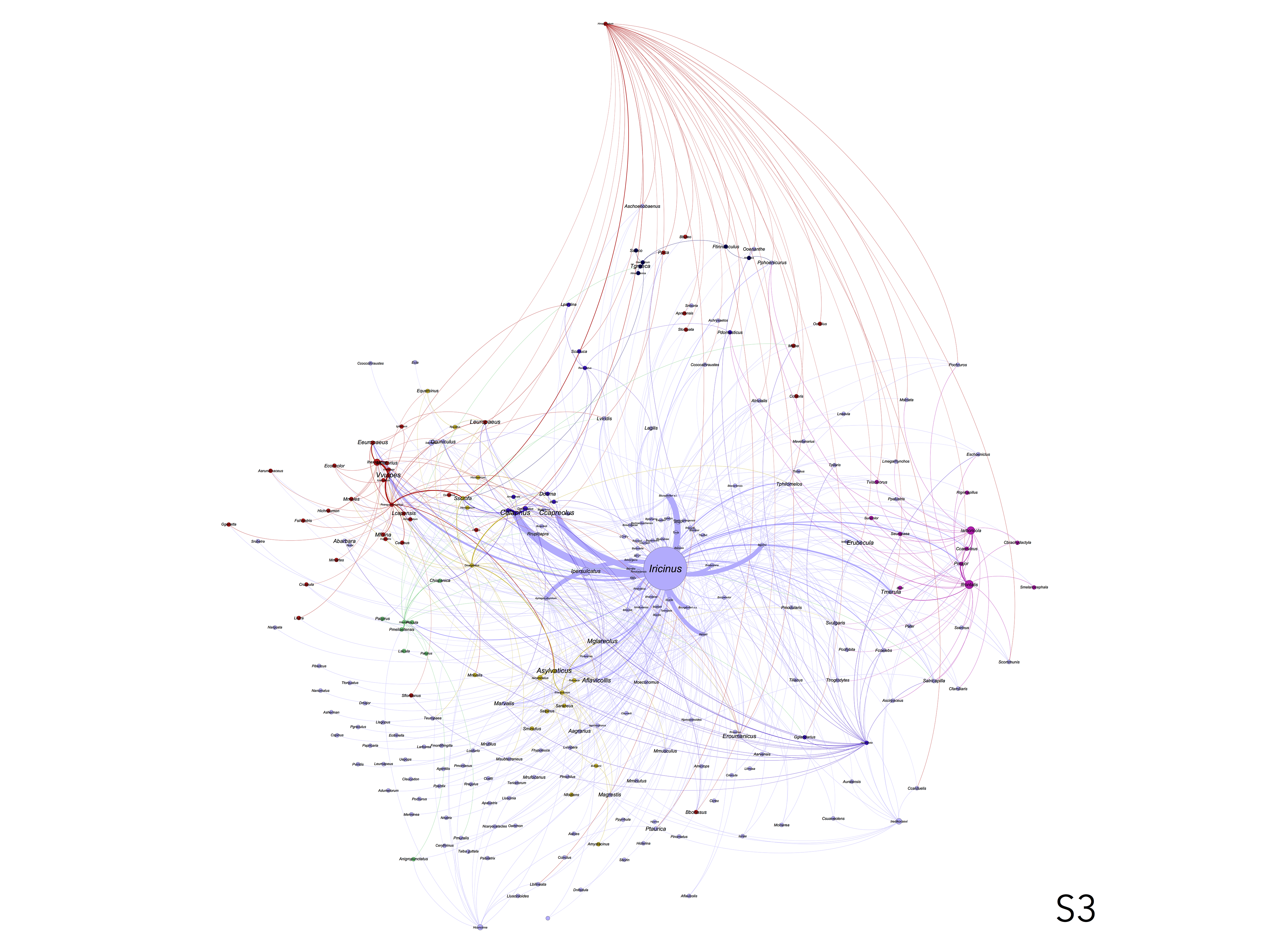

Supplement: Supplementary Figure S3 — The network of tick I. ricinus, the satellite species of ticks, vertebrate hosts, and transmitted pathogens. Circles are organisms (ticks, hosts, pathogens) and lines are links (interactions) among them. Colors in the figure indicate clusters retrieved by an algorithm that groups together the organisms that are closer than others. Clusters include other species of ticks that are satellite to the focal species because they share hosts. The size of each circle indicates the centrality of the organism. The width of each link is proportional to the strength of the interaction between two given organisms. [file Image3.TIFF]
