# Supplementary material for: Functional Redundancy and Ecological Innovation Shape the Circulation of Tick-Transmitted Pathogens
Source: Front Cell Infect Microbiol. 2017 May 31;7:234. doi: 10.3389/fcimb.2017.00234 (PMC5450623; doi:10.3389/fcimb.2017.00234)
Supplement: Supplementary Table S1 — The tree in newick format used to draw the phylogenetic relationships between the vertebrates recorded as hosts of Ixodes ricinus. [file DataSheet1.PDF]

(((((Micromys_minutus:28.93076321,(((Apodemus_mystacinus:9.240080369,Apodemus_agrarius:9.240
080369):6.633770398,((Apodemus_sylvaticus:1.728338794,Apodemus_uralensis:1.728338794):3.07166
1206,Apodemus_flavicollis:4.8):11.07385077):12.72930305,Mus_musculus:28.60315381):0.3276093938
):18.9268641,(((Microtus_oeconomus:8.267780815,(Microtus_subterraneus:8.207523195,Microtus_agr
estis:8.207523196):0.06025761931):0.4577321995,Microtus_arvalis:8.725513015):10.73727782,Myode
s_glareolus:19.46279083):28.39483647):34.74237269,(Sciurus_vulgaris:73.8,((Dryomys_nitedula:32.9,El
iomys_quercinus:32.9):3.7,Muscardinus_avellanarius:36.6):37.2):8.8):6.4,((Lepus_europaeus:9.9056465
39,Lepus_capensis:9.905646539):14.19435346,Oryctolagus_cuniculus:24.1):64.9):7.2,((((Rupicapra_rupi
capra:26.69792918,(Capreolus_capreolus:17.7,(Cervus_elaphus:12.79013335,Dama_dama:12.79013335
):4.909866652):8.997929184):38.90207082,Sus_scrofa:65.6):19.8044267,((Felis_silvestris:53,(Genetta_g
enetta:31.8,Herpestes_ichneumon:31.8):21.2):11.9,((((Lutra_lutra:11.5,((Mustela_nivalis:5.3,Mustela_p
utorius:5.3):1.3,Mustela_erminea:6.6):4.9):3.2,(Martes_foina:2.3,Martes_martes:2.3):12.4):2.3,Meles_
meles:17):44.2,((Canis_aureus:2.6,Canis_lupus:2.6):13.5,Vulpes_vulpes:16.1):45.1):3.7):20.5044267):3.0
95573303,(Talpa_europaea:80.99149009,(Erinaceus_europaeus:75.4,(((Crocidura_suaveolens:10.04400
922,Crocidura_russula:10.04400922):0.3409420276,Crocidura_leucodon:10.38495125):36.21504875,((S
orex_alpinus:23.8,(Sorex_araneus:20.1,Sorex_minutus:20.1):3.7):18.2,(Neomys_fodiens:10.6,Neomys_a
nomalus:10.6):31.4):4.6):28.8):5.591490092):7.508509908):7.7):227.8,(((Psammodromus_algirus:100.39
5469,(((Lacerta_bilineata:11.36925,Lacerta_viridis:11.36925):16.709495,Lacerta_agilis:28.078746):25.03
3346,(Zootoca_vivipara:48.471427,(Podarcis_sicula:24.977408,Podarcis_muralis:24.977407):23.494019)
:4.640665):47.283378):106.231061,Natrix_natrix:206.62653):93.37347,((((((Aquila_chrysaetos:50.1142
12,(Buteo_buteo:43.766174,Accipiter_gentilis:43.766174):6.348039):60.793196,(((Dendrocopos_major:
36.253052,Picus_viridis:36.253052):58.046418,Upupa_epops:94.29947):12.95208,(Strix_aluco:96.04669
7,Tyto_alba:96.046697):11.204853):3.655859):0.636515,(((Oriolus_oriolus:60.93435,(Lanius_collurio:51
.404878,((Pica_pica:32.260168,(Nucifraga_caryocatactes:29.440385,Garrulus_glandarius:29.440385):2.8
19783):13.234395,Pyrrhocorax_graculus:45.494563):5.910315):9.529472):9.951811,((((Muscicapa_stria
ta:38.13537,(Erithacus_rubecula:33.496291,(Ficedula_hypoleuca:30.376442,((Luscinia_luscinia:3.24814
5,Luscinia_megarhynchos:3.248145):25.572551,((Phoenicurus_ochruros:8.654543,Phoenicurus_phoenic
urus:8.654543):18.017153,(Oenanthe_oenanthe:21.617948,Saxicola_rubetra:21.617949):5.053747):2.1
49001):1.555746):3.119849):4.639079):10.666957,(Cinclus_cinclus:45.384915,(Turdus_viscivorus:16.71
5512,((Turdus_merula:10.316287,((Turdus_pilaris:6.442869,Turdus_torquatus:6.442869):2.165484,Turd
us_iliacus:8.608353):1.707934):5.362994,Turdus_philomelos:15.679281):1.036231):28.669403):3.41741
1):11.38573,((Regulus_ignicapillus:12.513646,Regulus_regulus:12.513646):44.885224,(Sitta_europaea:5
1.506649,(Troglodytes_troglodytes:46.8162,(Certhia_brachydactyla:3.717616,Certhia_familiaris:3.7176
16):43.098584):4.690448):5.892221):2.789186):3.068811,(((Parus_major:11.190673,(Parus_ater:9.0408
67,(Parus_caeruleus:7.608687,Parus_palustris:7.608686):1.43218):2.149806):48.215678,((Lullula_arbor
ea:24.70809,Alauda_arvensis:24.708089):30.317861,(((((Phylloscopus_sibilatrix:16.94406,(Phylloscopus
_collybita:8.251487,Phylloscopus_trochilus:8.251487):8.692573):1.719542,Phylloscopus_inornatus:18.6
636):29.006687,Cettia_cetti:47.670289):3.500051,(Locustella_naevia:49.513514,((Sylvia_atricapilla:8.65
5284,Sylvia_borin:8.655284):2.507454,(((Sylvia_melanocephala:4.374953,Sylvia_communis:4.374953):1
.723853,Sylvia_curruca:6.098806):1.308912,Sylvia_nisoria:7.407718):3.755019):38.350776):1.656825):2
.485425,((Acrocephalus_schoenobaenus:4.314365,(((Acrocephalus_scirpaceus:2.549794,Acrocephalus_
palustris:2.549794):0.885918,Acrocephalus_dumetorum:3.435712):0.587317,Acrocephalus_arundinace
us:4.023029):0.291335):16.50318,Hippolais_icterina:20.817544):32.83822):1.370186):4.3804):2.570182
,(((Passer_montanus:9.666265,Passer_domesticus:9.666265):35.864938,((((Emberiza_citrinella:15.5567

62,Emberiza_cia:15.556762):7.076494,Emberiza_schoeniclus:22.633255):17.722038,((Fringilla_coelebs:9.103021,Fringilla_montifringilla:9.103021):27.168132,((Carpodacus_erythrinus:25.594215,(Carduelis_chloris:19.487631,(Serinus_serinus:14.604686,Carduelis_carduelis:14.604686):4.882946):6.106584):2.974182,Pyrrhula_pyrrhula:28.568397):7.702756):4.08414):3.085708,((Anthus_trivialis:11.986203,Anthus_pratensis:11.986203):18.431611,(Motacilla_cinerea:3.729084,Motacilla_alba:3.729083):26.68873):13.023187):2.090203):4.776835,Prunella_modularis:50.308039):11.668493):1.280335):7.629294):34.053763,Falco_tinnunculus:104.939923):6.604):1.386727,(Numenius_arquata:99.335223,(Pluvialis_apricaria:68.764617,Vanellus_vanellus:68.764616):30.570607):13.595428):1.149218,Crex_crex:114.079869):11.920131,((Perdix_perdix:49.123371,Lagopus_lagopus:49.123372):7.938988,Alectoris_barbara:57.062358):68.93764):132.884783,Testudo_graeca:258.884783):41.115215):24);
